# Automated Detection of Gastrointestinal Diseases Using Resnet50*-Based Explainable Deep Feature Engineering Model with Endoscopy Images

**DOI:** 10.3390/s24237710

**Published:** 2024-12-02

**Authors:** Veysel Yusuf Cambay, Prabal Datta Barua, Abdul Hafeez Baig, Sengul Dogan, Mehmet Baygin, Turker Tuncer, U. R. Acharya

**Affiliations:** 1 Department of Digital Forensics Engineering, Technology Faculty, Firat University, Elazig 23119, Türkiye; 231144201@firat.edu.tr (V.Y.C.); turkertuncer@firat.edu.tr (T.T.); 2 Department of Electrical and Electronics Engineering, Faculty of Engineering and Architecture, Mus Alparslan University, Mus 49250, Türkiye; 3 School of Business (Information System), University of Southern Queensland, Toowoomba, QLD 4350, Australia; prabal.barua@usq.edu.au; 4 School of Management and Enterprise, University of Southern Queensland, Toowoomba, QLD 4350, Australia; abdul.hafeez-baig@unisq.edu.au; 5 Department of Computer Engineering, Faculty of Engineering and Architecture, Erzurum Technical University, Erzurum 25500, Türkiye; mehmet.baygin@erzurum.edu.tr; 6 School of Mathematics, Physics and Computing, University of Southern Queensland, Springfield, QLD 4300, Australia; rajendra.acharya@usq.edu.au

**Keywords:** ResNet50*, colon disease classification, deep feature engineering, multiple iterative feature selection

## Abstract

This work aims to develop a novel convolutional neural network (CNN) named ResNet50* to detect various gastrointestinal diseases using a new ResNet50*-based deep feature engineering model with endoscopy images. The novelty of this work is the development of ResNet50*, a new variant of the ResNet model, featuring convolution-based residual blocks and a pooling-based attention mechanism similar to PoolFormer. Using ResNet50*, a gastrointestinal image dataset was trained, and an explainable deep feature engineering (DFE) model was developed. This DFE model comprises four primary stages: (i) feature extraction, (ii) iterative feature selection, (iii) classification using shallow classifiers, and (iv) information fusion. The DFE model is self-organizing, producing 14 different outcomes (8 classifier-specific and 6 voted) and selecting the most effective result as the final decision. During feature extraction, heatmaps are identified using gradient-weighted class activation mapping (Grad-CAM) with features derived from these regions via the final global average pooling layer of the pretrained ResNet50*. Four iterative feature selectors are employed in the feature selection stage to obtain distinct feature vectors. The classifiers k-nearest neighbors (kNN) and support vector machine (SVM) are used to produce specific outcomes. Iterative majority voting is employed in the final stage to obtain voted outcomes using the top result determined by the greedy algorithm based on classification accuracy. The presented ResNet50* was trained on an augmented version of the Kvasir dataset, and its performance was tested using Kvasir, Kvasir version 2, and wireless capsule endoscopy (WCE) curated colon disease image datasets. Our proposed ResNet50* model demonstrated a classification accuracy of more than 92% for all three datasets and a remarkable 99.13% accuracy for the WCE dataset. These findings affirm the superior classification ability of the ResNet50* model and confirm the generalizability of the developed architecture, showing consistent performance across all three distinct datasets.

## 1. Introduction

The gastrointestinal (GI) system is the digestive system, which contains organs and tissues responsible for digestion, absorption of nutrients, and elimination of waste from the body [1,2]. The first organ of the GI system is the mouth, and it concludes with the expulsion of feces from the anus [3]. Each organ in the GI system has its specific function: the mouth initiates digestion through mechanical and chemical processes; acidic gastric juices in the stomach break down food and prepare it for the small intestine. The large intestine absorbs beneficial fluids from the food, delivers waste to the anus via feces, and expels waste [4]. The GI system is prone to various diseases [5], and advanced technologies have been employed in recent years for their automated diagnosis [6,7,8,9]. With the emergence of advanced imaging technologies like endoscopy and colonoscopy, a vast amount of GI images related to the GI have been developed [10]. Artificial intelligence (AI)-based classification systems contribute to developing automatic classification models using these images [11,12]. These AI-supported systems are used to identify patterns and abnormalities in gastrointestinal images, ranging from polyps to ulcers, tumors to lesions, with high accuracy and efficiency. Training on extensive datasets of annotated GI images enables these systems to visualize subtle visual structures in various GI conditions, facilitating early detection and intervention [13]. Additionally, AI-supported GI image classification aims to streamline the diagnostic process, reduce the burden on healthcare providers, and thus improve patient outcomes through timely and accurate medical interventions [14].

### 1.1. Literature Review

There are various AI-based systems developed for the detection of different diseases in the literature [15,16,17,18]. The AI methods used to detect GI diseases are presented in this section. Caroppo et al. [19] introduced deep transfer learning approaches for bleeding detection in endoscopy images. Their study utilized two benchmark datasets and employed a feature selection fusion approach to optimize the feature sets for classification, and they achieved accuracy rates of 97.65% and 95.70% on these datasets. Their study demonstrated the effectiveness of transfer learning in detecting bleeding lesions using endoscopy images. Ghosh and Chakareski [20] proposed a novel approach for identifying bleeding in endoscopic images. Their study demonstrated significant improvements over existing methods and reported a bleeding frame detection F1-score of 98.49% and a bleeding zone detection accuracy of 94.42%. Zhang et al. [21] explored deep transfer learning from ordinary to capsule esophagogastroduodenoscopy for image quality control. Using a dataset of 62,850 capsules and 17,434 ordinary endoscopy images, the dynamic adversarial adaptation network achieved an AUROC of 0.8638 in internal cross-validation and 0.9471 in prospective validation, outperforming conventional CNNs and vision transformers (ViTs). Lonseko et al. [22] introduced attention-guided CNNs for gastrointestinal disease classification in endoscopic images. Their results demonstrated superior performance compared with other state-of-the-art models, with mean accuracies reported for various models (ResNet50 = 90.28%, GoogLeNet = 91.38%, Dense Convolutional Network (DenseNet) = 91.60%, and their baseline model achieving 92.84%). The proposed method achieved a precision of 92.8%, recall of 92.7%, F1-score of 92.8%, and overall accuracy of 93.19%, showcasing the potential of attention-guided networks in medical image analysis for GI disease classification. Their attention mechanism enhanced model interpretability and classification accuracy, contributing to developing robust computer-aided diagnosis systems for GI diseases. He et al. [23] proposed a deep learning-based anatomical site classification for upper gastrointestinal endoscopy. By acquiring 5661 esophagogastroduodenoscopy (EGD) images from 229 clinical cases, the research team developed and annotated a dataset following a modified guideline that integrates British and Japanese standards for endoscopic documentation. Their work focused on accurate anatomical site localization, showcasing the potential of deep learning in facilitating precise lesion localization and diagnosis. Subedi et al. [24] proposed a hybrid CNN–transformer model to enhance the classification of GI, leveraging the strengths of DenseNet201 for local feature extraction and the Swin Transformer for global context analysis. Using the GastroVision and Kvasir-Capsule datasets, which include endoscopic and video capsule images, the model achieved superior performance metrics, with a Matthews Correlation Coefficient (MCC) of 0.8191 for the GastroVision dataset and 0.3871 for the Kvasir-Capsule dataset, surpassing standalone CNN and Swin Transformer models. Patel et al. [25] developed a deep learning-based approach for classifying GI diseases using pre-trained CNNs with transfer learning. Their study utilized the Kvasir dataset, comprising 4000 labeled endoscopic images across 8 classes, to evaluate the performance of the models. EfficientNetB5 achieved the highest testing accuracy, 92.58%, with precision, recall, and F1-score all reaching 93%. Huo et al. [26] proposed the HiFuse model, a hierarchical multi-scale feature fusion network designed for medical image classification, addressing challenges like intra-class variation and inter-class similarity in medical imaging. Their study utilized Kvasir. The HiFuse model achieved high classification performance, with accuracy and F1-scores reaching 86.12% and 86.13%, respectively, on Kvasir.

### 1.2. Literature Gaps

Based on our literature review, we have identified several gaps in the research, which are summarized below:

- The current literature employed well-established convolutional neural networks (CNNs). Hence, there is a scarcity of innovative CNNs or deep learning models in this field.
- Explainable artificial intelligence (XAI) is a significant branch of machine learning that offers insights into the learning of deep learning models. However, in the realm of biomedical image classification, there are few XAI models available. This is mainly because the existing models focus predominantly on classification results rather than providing an understanding of the learning process.
- The models developed typically employed either deep learning or feature engineering methodologies but not a combination of both.
- A majority of the advanced models have been evaluated using only a single dataset, which may limit their generalizability to diverse scenarios.

### 1.3. Motivation and Study Outline

This work aims to contribute to deep learning and feature engineering. It is well established in the literature that Residual Networks (ResNets) are efficient CNNs, and recently, transformers have emerged as the forefront technology in computer vision [27]. This work is focused on developing an effective and explainable model for medical applications. Self-organized models represent the next wave in machine learning, yet such models are scarce in the existing literature.

Hence, we introduced an enhanced version of ResNet, ResNet50*, which is attention-based, drawing inspiration from PoolFormer [28]. We have incorporated convolution-based residual blocks to tackle the issue of vanishing gradients. Using the pretrained ResNet50* and Grad-CAM [29], we have segmented the region of interest (ROI) for feature extraction, thereby generating meaningful features through XAI.

We have also developed a self-organized DFE model that utilizes multiple iterative feature selectors, various classifiers, and an information fusion approach. This DFE model employed ResNet50*, integrating deep learning and feature engineering. The self-organized nature of the proposed DFE, based on ResNet50*, is the main novelty of this work. We have developed this ResNet50* model using three different image datasets.

### 1.4. Novelties and Contributions

Our proposed model introduces several novel features and significantly contributes to the field. The innovations and contributions of the proposed ResNet50* are given below:

Novelties:
  - We have introduced a novel ResNet50*-based explainable DFE model, which yielded the highest classification performance using three datasets.
  - We used three datasets to develop the ResNet50*-based explainable deep feature engineering model.
Contributions:
  - ResNet models are widely recognized in computer vision. This work proposes a new ResNet model, ResNet50*, to improve the classification capabilities of the original ResNet50. This model incorporates a convolution-based residual block and a pooling-based attention algorithm. Additionally, we have developed a self-organized DFE model based on ResNet50*, which is a significant contribution to both deep learning and feature engineering.
  - The proposed DFE model has been rigorously tested using three distinct datasets. Our model has demonstrated high classification accuracy across all these image datasets, confirming its effectiveness. Importantly, the proposed DFE model is also explainable, enhancing its value in practical applications.

## 2. Materials and Methods

### 2.1. Material

**Figure 1:**
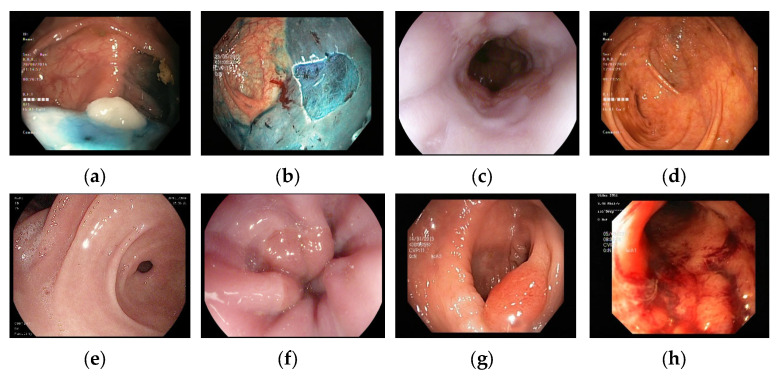
Sample images used in this work. (**a**) Dyed lifted polyps. (**b**) Dyed resection margins. (**c**) Esophagitis. (**d**) Normal cecum. (**e**) Normal pylorus. (**f**) Normal z-line. (**g**) Polyps. (**h**) Ulcerative colitis.

Three gastrointestinal image (GI) datasets were used: (i) Kvasir, (ii) wireless capsule endoscopy (WCE), and (iii) Kvasir v2, containing 8, 4, and 8 classes, respectively. The training images from the Kvasir dataset were augmented, and the newly developed ResNet50* was trained with this augmented dataset. This augmented dataset was then applied to obtain a pretrained version of ResNet50*. Using this pretrained ResNet50*, the deep feature engineering (DFE) model was developed. Sample images from these datasets are shown in Figure 1.

#### 2.1.1. Kvasir Dataset

The primary dataset employed in our study is the Kvasir dataset [30,31]. This dataset comprises eight distinct classes, and we augmented the training images within this dataset to train the pretrained version of ResNet50* to develop an explainable DFE model. The details of the Kvasir dataset, including the different classes and the numbers of training and testing images, is presented in Table 1.

The Kvasir dataset, along with the Kvasir v2 and WCE datasets, are widely used, publicly available datasets in the research community for gastrointestinal image analysis. In this study, the choice of using 97% of the Kvasir dataset for training and 3% for testing is motivated by the need for a large dataset to train the proposed ResNet50*-based DFE model, allowing the ResNet50* to be trained robustly and enabling it to learn diverse features. The small percentage allocated to the test set allows the study to leverage the high-quality features extracted by the pretrained ResNet50*. The ResNet50* model used in this study is pretrained on the augmented Kvasir dataset. This pretrained ResNet50* model is then used to extract features from other datasets (Kvasir v2 and WCE) without requiring additional training. The Kvasir v2 dataset, consisting of 8000 images, and the WCE dataset, containing 4 balanced image classes, are used to evaluate the generalizability and effectiveness of the proposed model.

#### 2.1.2. WCE Dataset

The WCE dataset was downloaded from Kaggle [32] and contains four classes: (i) normal, (ii) ulcerative colitis, (iii) polyps, and (iv) esophagitis. This dataset is balanced, like the Kvasir dataset, and uses the test folder containing 200 images in each class.

#### 2.1.3. Kvasir Version 2 Dataset

This dataset is the second version of the Kvasir dataset and contains eight classes, like the original Kvasir dataset [33]. There are 8000 images in this dataset, with 1000 images in each class.

### 2.2. ResNet50*

A new CNN model named ResNet50*, with an explainable deep feature engineering model, is proposed for gastrointestinal disease detection using endoscopic images. The small capsule-sized devices known as WCE sensors are equipped with cameras to capture detailed images as the capsule passes through the digestive system after being swallowed by the patient. These sensors can be transformed into smart sensors when used with ResNet50* to diagnose conditions such as ulcers, polyps, and inflammatory diseases like ulcerative colitis. The performance of the proposed ResNet50* model depends on the quality of the data captured by the sensors.

The proposed ResNet50* model is likely to yield higher classification performance by integrating convolution-based residual blocks and a pooling-based attention mechanism. ResNet50 was selected as the foundation for this model because it is a widely used CNN. However, with the advent of transformers, which use attention mechanisms, CNNs have been less widely used. To address this gap, a novel mechanism that combines convolution- and pooling-based attention is introduced.

**Figure 2:**
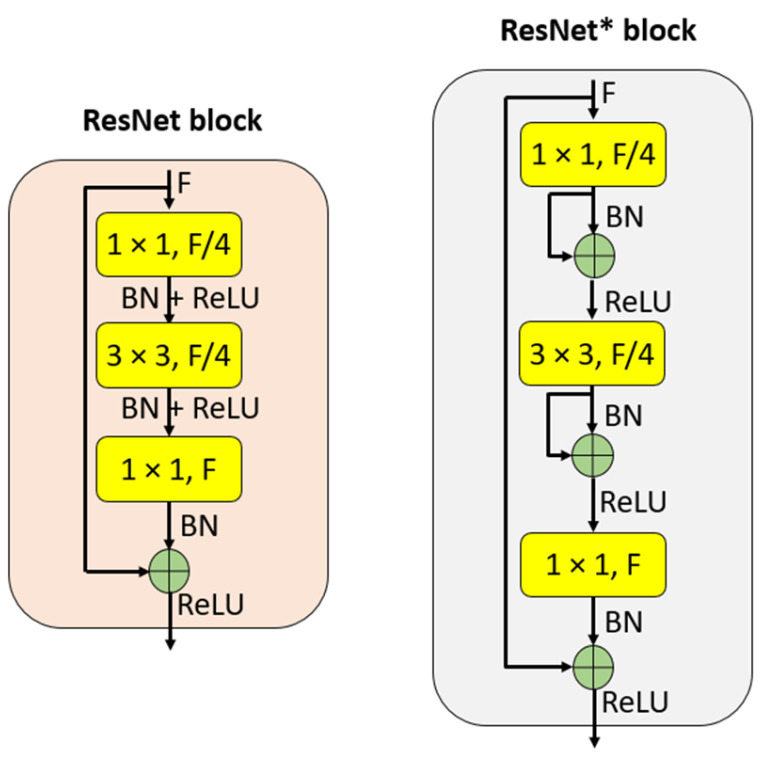
Block designs for ResNet and ResNet*. F: number of filters.

We demonstrated how ResNet50* differs from the original ResNet50 [34] in Figure 2, using the main blocks of CNN. This highlights the enhancements and innovations that ResNet50* brings to the conventional CNN architecture, particularly in terms of attention mechanisms.

**Figure 3:**
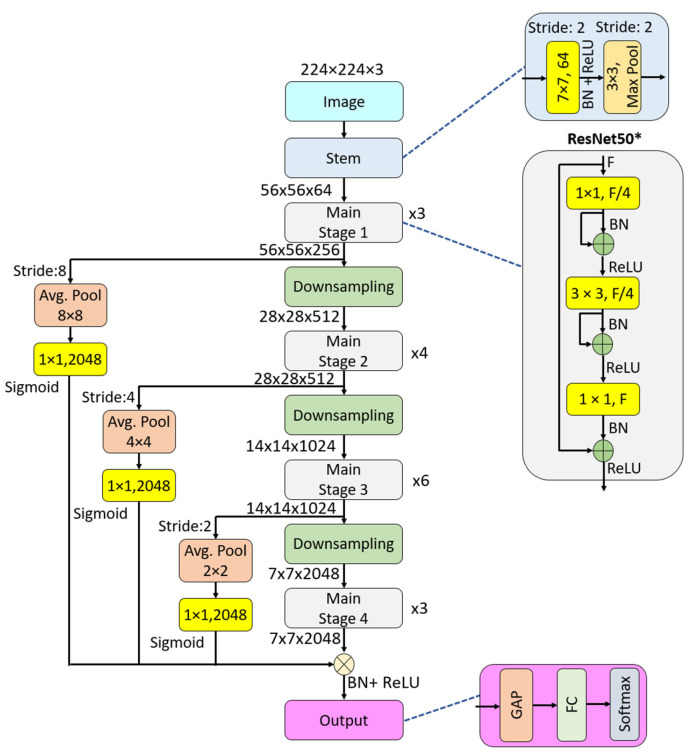
Graphical demonstration of the proposed ResNet50*. F: number of filters, BN: batch normalization, ReLU: restricted linear unit, Avg. Pool: average pooling, Max Pool: maximum pooling, GAP: global average pooling, FC: fully connected.

Figure 2 depicts the block designs for both ResNet and ResNet*. It is evident from the figure that we have implemented a convolution-based residual block in the ResNet* blocks. Using these ResNet* blocks, we developed ResNet50*, and its graphical representation is shown in Figure 3.

Figure 3 illustrates that the proposed model incorporates both average pooling and a convolution-based attention mechanism. The average pooling functions as patchify blocks, while pixel-wise convolution blocks are used for scaling.

The pseudocode of the ResNet50* model is given below.

In the ResNet50* block (see Algorithm 1), shortcuts are enhanced by using the convolution-based residual block. In the pooling-based attention layer, the outputs of all stages are scaled and added to the feature map creation stage.
**Algorithm 1.** Pseudocode of the ResNet50* model.**Input:** Image of size of 224 × 224 × 3 **Output:** Class probabilities01: Stem Block:   Apply 7 × 7 convolution with stride 2, F = 64  Apply Batch Normalization (BN) and ReLU  Apply 3 × 3 Max Pooling with stride 2 02: Stage 1:  Repeat ResNet* block 3 times with F = 256  (Each block contains 1 × 1 convolution, 3 × 3 convolution, 1 × 1 convolution with BN and ReLU between layers). 03: Stage 2:    Apply downsampling with stride 2   Repeat ResNet* block 4 times with F = 512 04: Stage 3:   Apply downsampling with stride 2    Repeat ResNet* block 6 times with F = 1024 05: Stage 3:   Apply downsampling with stride 2    Repeat ResNet* block 3 times with F = 2048 06: Attention Mechanism with Average Pooling and Scaling,   For each stage output (1–4):    Apply average pooling with appropriate size (8 × 8 for Stage 1, 4 × 4 for Stage 2, 2 × 2 for Stage 3, 1 × 1 for Stage 4)      Use 1 × 1 convolution to match the channel dimensions (2048)    Apply Sigmoid activation for scaling 07: Final Layers:   Apply Batch Normalization and ReLU    Apply Global Average Pooling (GAP)    Fully Connected (FC) layer for classification   Softmax activation for output

Overall, ResNet50* is designed to improve the capabilities of ResNet50, particularly addressing the limitations related to attention mechanisms found in transformers. ResNet50* aims to deliver superior classification performance in various computer vision tasks by integrating convolution-based attention with pooling mechanisms.

## 3. The Presented Deep Feature Engineering Model Based on ResNet50*

The second model we proposed is an explainable self-organized deep feature engineering (DFE) model. This model aims to achieve high test classification performance. The DFE model encompasses four main phases: (i) deep feature extraction using Grad-CAM and the global average pooling (GAP) layer of the pretrained ResNet50*, (ii) feature selection with four iterative feature selectors, (iii) classification using kNN [35] and SVM [36] classifiers, and (iv) information fusion.

In the first phase, the pretrained ResNet50* is employed to identify regions of interest (ROIs) in images. Grad-CAM is applied to create a heatmap for each image, which helps segment the ROI. Features are then extracted from this segmented area using the GAP layer of the pretrained ResNet50*, resulting in feature vectors of length 2048.

During the feature selection phase, four iterative feature selectors are utilized: (i) Iterative Neighborhood Component Analysis (INCA) [37], (ii) Iterative Chi-squared (IChi2) [38], (iii) Iterative Minimum Redundancy Maximum Relevance (ImRMR) [39], and (iv) Iterative ReliefF (IRF) [40]. These selectors help to generate four distinct sets of selected feature vectors.

For classification, the selected feature vectors are processed using kNN and SVM classifiers, producing a total of 8 (=4 × 2) classifier-based outcomes.

In the information fusion phase, iterative majority voting (IMV) [41] creates an additional 6 voted outcomes, resulting in a total of 14 (8 + 6) outcomes. The best outcome is then selected through a greedy algorithm.

**Figure 4:**
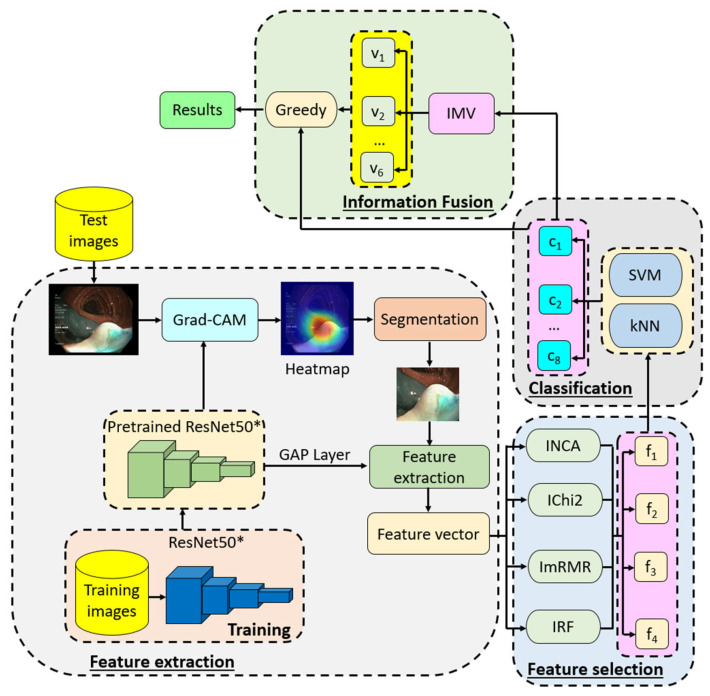
Graphical overview of the proposed ResNet50*-based DFE model. Here, f: selected feature vector, c: classifier-based outcome, v: voted outcome.

A schematic diagram of the proposed DFE model is presented in Figure 4.

To better explain the proposed model, we present the steps of the presented DFE model.

Step 1: Train the ResNet50* by deploying training images of the Kvasir image dataset and obtain the pretrained ResNet50*.

Step 2: Load test images.

Step 3: Apply Grad-CAM to each test image and generate a heatmap.

Step 4: Segment the hot areas of the image. In this step, we use the read areas as ROI, and the features are extracted from these areas.

Step 5: Extract deep features by deploying the GAP layer of the pretrained ResNet50*.

The five steps above have been defined as the proposed explainable deep feature extraction phase of the proposed ResNet50*-based DFE model. The feature selection steps are defined below.

Step 6: Select the most informative features by deploying iterative feature selectors. These feature selectors are (i) INCA, (ii) IChi2, (iii) ImRMR, and (iv) IRF. We have used an iterative feature selection structure, and the pseudocode of the iterative feature selection is explained in Algorithm 2.
**Algorithm 2.** Iterative feature selection**Input:** Feature matrix (X), actual output (y), the used feature selector ($fsel\left(.,.\right)$), the used loss value generator (L(.,.)). **Output:** The selected feature vector (s).01: index=fsel(X,y); //Here, index is the qualified indices of the feature matrix. //Generate the qualified indices of the features by deploying the used feature selection function. 02: **for** i = 0 to fv−sv **do**//Select features iteratively. Herein, sv: start value, fv: finite value. 03:   **for** j = 1 to sv+i **do** 04:      sf(:,j)=X(:,index(j));
05:   **end for** j 06:    loss(i+1)= L(sf,y); //Calculate loss values of the each selected feature vector. 07:   id=min(loss); //Compute id of the minimum loss value. 08: **end for** i 09: **for** k = 1 to sv+id−1 **do** 10:   s(:,k)=X(:,index(k)); //Create the final feature vector. 11: **end for** k

As seen from Algorithm 2, iterative feature selectors are used differently to select the best-selected feature vectors.

Step 7: Classify the selected *four* feature vectors by deploying kNN and SVM. The first four classifier-based outcomes are generated by deploying the kNN classifier, and the remaining four outcomes are created by the SVM classifier. Therefore, 8 classifier-based outcomes are created in this step. For each feature vector, classification is performed using kNN and SVM models. The kNN classifier produces 4 outcomes (one for each feature vector), and the SVM classifier also generates 4 outcomes, resulting in a total of 8 classifier-based outcomes.

To ensure robust performance, 10-fold cross-validation is used to train and evaluate both kNN and SVM classifiers. The pretrained ResNet50* model is used for feature extraction, and the resulting feature vectors are used as input for these classifiers. This training process ensures the generalizability of the classifiers and their ability to accurately distinguish between classes.

Step 8: Produce voted outcomes utilizing the IMV algorithm. IMV was proposed by Dogan et al. [41]. We explain the IMV algorithm in Algorithm 3.
**Algorithm 3.** Procedure of the IMV.**Input:** Classifier-based outcomes (c), real output (y). **Output:** Voted outcomes (v).01: $acc(t)=\alpha \left(c_{t},y\right),\;t\in \{1,2,\ldots ,8\}$
//Here, acc is the computed classification accuracy by deploying the classification accuracy calculation function (α(.,.)). 02: ind=S(acc); //Sort the classification accuracies from high to low and ind is the qualified index. where S(.) is the sorting function. 03: **for** i = 3 to 8 **do** //Apply iterative majority voting. 04:   **for** j = 1 to D **do** //Herein, D is the number of images 05:    **for** k = 1 to i **do** 06:     $arr\left(k\right)=c_{ind\left(i\right)}(j)$; //Create array (arr). 07:    **end for k** 08:    $v_{i-2}\left(j\right)=\varpi (arr)$
//Apply mode (ϖ(.)) function to the created array to generate a voted value 09:   **end for j**
10: **end for i**


As presented in Algorithm 3, we have generated the six voted outcomes by deploying the eight classifier-based outcomes.

Step 9: Using a greedy algorithm, the best outcome among the created 14 (=8 classifier-based + 6 voted) outcomes per classification accuracy is obtained.

(1) $$\begin{array}{l} acc(t)=\alpha \left(c_{t},y\right),\;t\in \left\{1,2,\ldots ,8\right\}\\ acc\left(8+h\right)=\alpha \left(v_{h},y\right),\;w\in \left\{1,2,\ldots ,6\right\}\\ ind=\max\left(acc\right)\\ result=\left\{\begin{array}{c} c_{ind},ind\leq 8\\ v_{ind-8},\;id>8 \end{array}\right. \end{array}$$

Herein, result is the final outcome.

During the selection of the best result in the proposed ResNet50*-based deep feature engineering model, accuracy plays a crucial role and is calculated using 10-fold cross-validation in the training phase, and test results are computed. For the 8 classifier-based results (4 from kNN and 4 from SVM) and 6 voted results (generated using the IMV algorithm), cross-validation ensures that the accuracy of each result is an unbiased and robust estimate of its performance. Using this cross-validated accuracy, a greedy algorithm selects the result with the highest accuracy and determines the single best outcome among the 14 results (8 classifier-based + 6 voted). This approach ensures that the selection process does not interfere with the evaluation of the testing phase, preserving the integrity of the results while utilizing the most reliable output.

## 4. Experimental Results

This section presents the classification performances obtained for the ResNet50*-based explainable deep feature engineering (DFE) model. ResNet50* and the DFE models were developed using the MATLAB (2023a) programming environment. To develop ResNet50*, the deep network designer tool available in MATLAB was used. We modified the existing ResNet50 CNN to tailor it to our requirements and created our proposed CNN model. To develop the presented DFE model, we employed MATLAB’s ‘m’ files, which are script files used to execute MATLAB commands and function sequences.

### 4.1. Experimental Settings

In this work, we trained the proposed ResNet50* and then developed a deep feature engineering (DFE) model based on this ResNet50*. To implement these models, we used the following parameters:

Training parameters for ResNet50*:

Solver: Stochastic Gradient Descent with Momentum (sgdm); Initial Learning Rate: 0.01; Mini-Batch Size: 128; Maximum Epochs: 25; L2 Regularization: 0.0001; training and validation split ratio: 80:20.

Parameters for the DFE based on ResNet50*:

The DFE model consists of four phases, and the parameters for each phase are as follows:

Feature extraction phase:

Image segmentation method: Grad-CAM; Mask Generation Method: Grad-CAM-based score map generation and thresholding; Threshold Value: mean value of the score map × √2; Feature extraction layer: global average pooling (GAP).

Feature Selection Phase:

Feature selectors used: NCA [42], Chi2 [43], mRMR [44], and RF [45]; Iteration range: 100 to 768; Loss value generator: kNN with 10-fold cross-validation; Final selected feature vector selection method: greedy algorithm.

Classification Phase:

kNN parameters: k: 10; Distance metric: Euclidean; Weight: squared inverse; Validation: 10-fold cross-validation.

SVM parameters: Kernel: third degree polynomial; Box constraint: 1; Coding: one-vs-all; Validation: 10-fold cross-validation.

Information fusion phase:

IMV parameters: Sorting criteria: Descending order based on classification accuracy; Iteration range: 3 to 8; Voting function: mode.

Greedy algorithm: Selection of one outcome with maximum accuracy.

Using these parameters, we successfully implemented both ResNet50* and DFE models.

### 4.2. Performance Analysis

We used (i) classification accuracy, (ii) F1-score, and (iii) geometric mean performance evaluation matrices.

**Figure 5:**
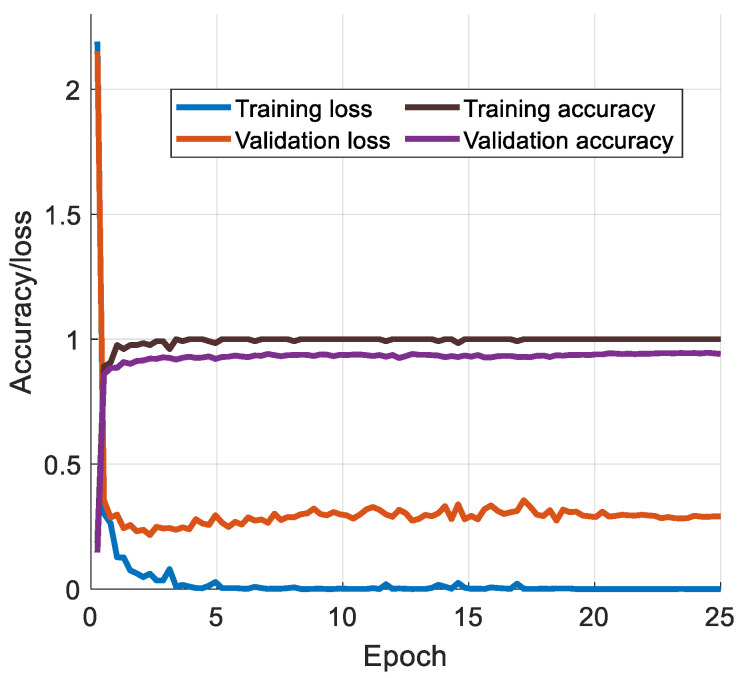
Graph of training and validation accuracies/losses versus number of epochs.

The training accuracy/loss versus the number of epochs for ResNet50* on the Kvasir dataset are in Figure 5.

Figure 5 shows that our model achieved 94.78% validation accuracy and a final loss value of 0.2603 with a maximum training accuracy of 100%.

Using the pretrained ResNet50*, we have developed and presented our DFE model. This DFE model is capable of generating outcomes based on both classifier-specific and voted approaches. Furthermore, we have applied three different datasets to this DFE model. We tested our DFE model on the test images of the Kvasir and WCE datasets, as these datasets share common images. The DFE model was fed with all images from the Kvasir v2 dataset. The summary of classification results obtained for the different datasets is summarized in Table 2.

Table 2 indicates that our proposed ResNet50*-based DFE model achieved test performance exceeding 92% for all the datasets. The highest results across all datasets were obtained from the voted outcomes. This finding strongly suggests that the IMV model significantly enhanced classification performance for all datasets. Our model is characterized as self-organized because it automatically selects the classification outcome with the best performance. Additionally, Table 2 includes a detailed tabulation of the classification performance of the proposed DFE model, which is further elaborated in Table 3.

**Figure 6:**
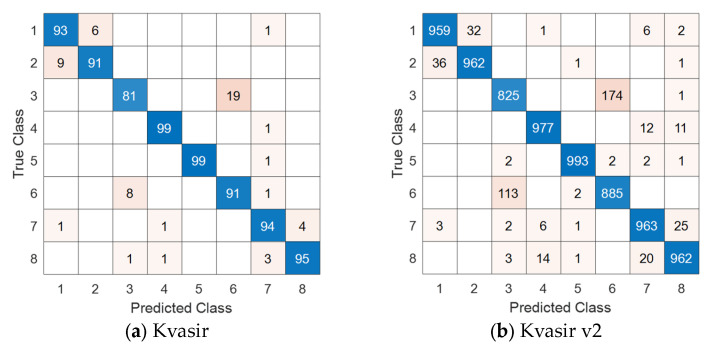

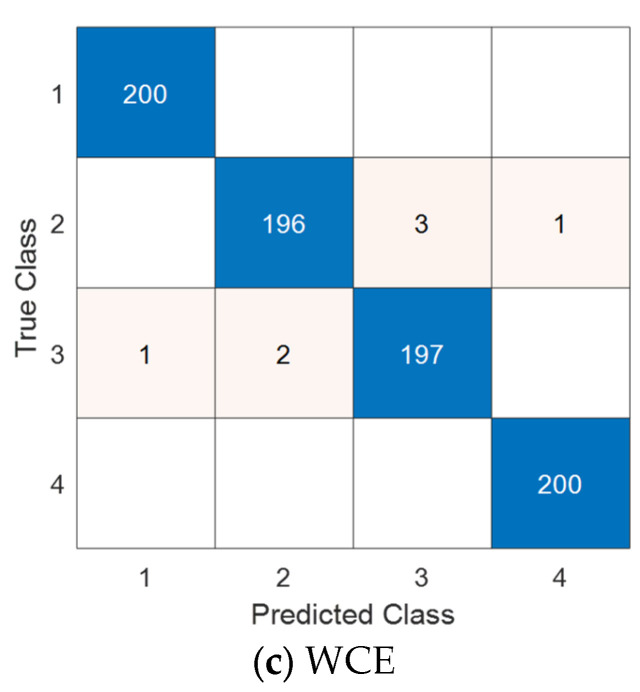
Confusion matrices obtained for the proposed ResNet50*-based DFE model. In the confusion matrices, cells with a blue background represent the correctly predicted observations (true positives) for each class. Cells with a white background represent zeros, indicating no predictions for those combinations. Cells with a beige background represent the number of falsely predicted observations (misclassifications) between classes.

Additionally, we have presented the computed confusion matrices for these datasets in Figure 6.

The numbers in the confusion matrices shown in Figure 6a,b correspond to the following classes: 1: Dyed lifted polyps; 2: Dyed resection margins; 3: Esophagitis; 4: Normal cecum; 5: Normal pylorus; 6: Normal z-line; 7: Polyps; and 8: Ulcerative colitis. In the confusion matrix for the WCE dataset (Figure 6c), four classes are represented: 1: Normal; 2: Ulcerative colitis; 3: Polyps; and 4: Esophagitis.

**Figure 7:**
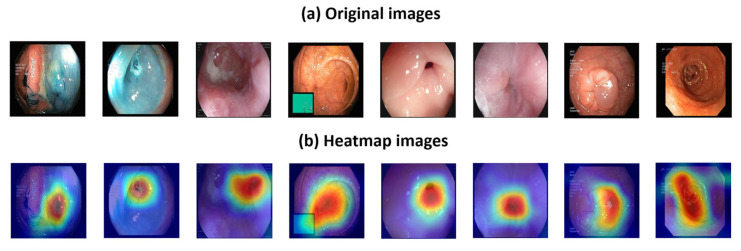
Sample images and their corresponding heatmaps. The colors in the heatmap typically represent varying levels of intensity or importance: blue tones indicate areas that are not important, yellow tones represent moderately important areas, and red tones highlight regions that are highly important for feature extraction.

Our model employed an attention-based convolutional neural network (CNN) and a Grad-CAM-based segmentation model to generate deep features from the images. The explainable results obtained are shown in Figure 7.

Figure 7 demonstrates that the proposed model is able to focus on the ROI accurately due to the attention mechanism employed in the ResNet50* model.

For all classes, the ROIs have been highlighted in red. The main findings are as follows:

- The red areas in each image represent the highest activation regions identified by Grad-CAM, indicating that the proposed ResNet50* model perceives these regions as the most relevant for classification. The yellow, green, and blue areas represent decreasing levels of importance.
- The use of Grad-CAM increases explainability and helps to understand why the model focuses on certain regions. This feature is particularly useful in medical imaging, where interpretability can aid clinical decisions and provide insights into how ResNet50* classifies.
- These images demonstrate how the presented ResNet50* can assist in detecting abnormalities (such as polyps, esophagitis, or ulcerative colitis) and in confirming normal anatomical structures. Focusing on ROIs supports professionals in making accurate diagnoses.

The results shown in Figure 7 highlight the ability of the model to locate ROIs in addition to its high classification performance. The focus on ROIs, enabled by the attention mechanism, leads to improved accuracy and reliability in classification and analysis, making the ResNet50* a valuable tool in advanced computer vision applications.

## 5. Discussion

**Figure 8:**
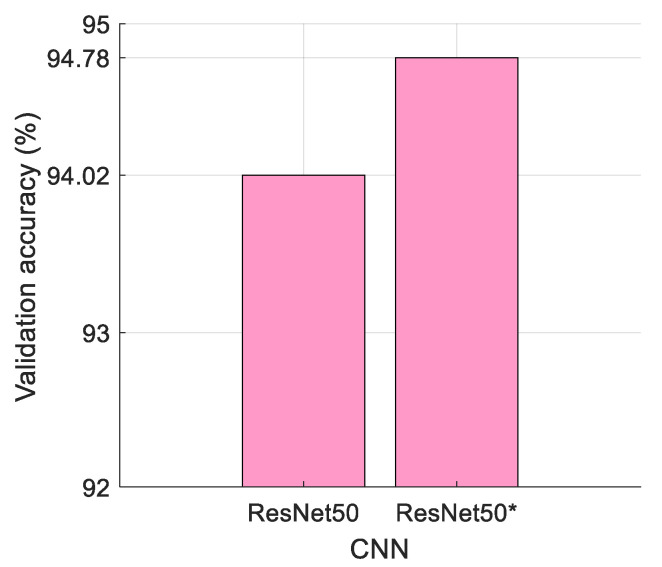
Validation accuracies (%) obtained for the ResNet50 and ResNet50* models.

The results obtained using our proposed models are discussed in this section. We present a novel ResNet50*-based model with about 28 million more parameters than ResNet. This increase in parameters is due to the inclusion of a pooling-based attention mechanism in ResNet50*. This mechanism does not increase the size of the pooling map, since we used multiplication before the output layer. The convolutions used for scaling have increased the number of parameters. This layer was incorporated to add attention to our proposed model and enhance the classification performance of the presented ResNet50*. Figure 8 presents the validation accuracies obtained using the ResNet50 and ResNet50* models.

It may be noted from the above figure that ResNet50* demonstrated a superior classification accuracy compared with ResNet50. Also, ResNet50* delineated ROI better, as shown in Figure 7. Hence, we chose ResNet50* over ResNet50 and introduced a novel DFE model with the pretrained ResNet50*. As shown in Figure 8, the performance comparison between the original ResNet50 model and the modified ResNet50* version was conducted using the pretrained ResNet50 model on the Kvasir dataset to provide a fair and unbiased evaluation. Both models were pretrained under identical conditions on the same dataset, ensuring comparability in terms of validation accuracy.

An ablation study was also conducted on the presented model. In this study, an attention block was used with ResNet50 in Case 1, and the ResNet* block was applied without pooling-based attention in Case 2. These results were evaluated on the Kvasir 2 dataset, yielding validation accuracies of 94.36% for Case 1 and 94.53% for Case 2. Furthermore, when these blocks were used together (to create ResNet50*), the computed validation accuracy increased to 94.78%.

The ResNet50*-based DFE model (this model is presented to increase the test classification accuracy) incorporated the INCA, IChi2, ImRMR, and IRF feature selectors along with kNN and SVM classifiers. This combination yielded eight classifier-based outcomes with six voted outcomes. The details of the feature selector and classifier combination are shown in Table 4.

**Figure 9:**
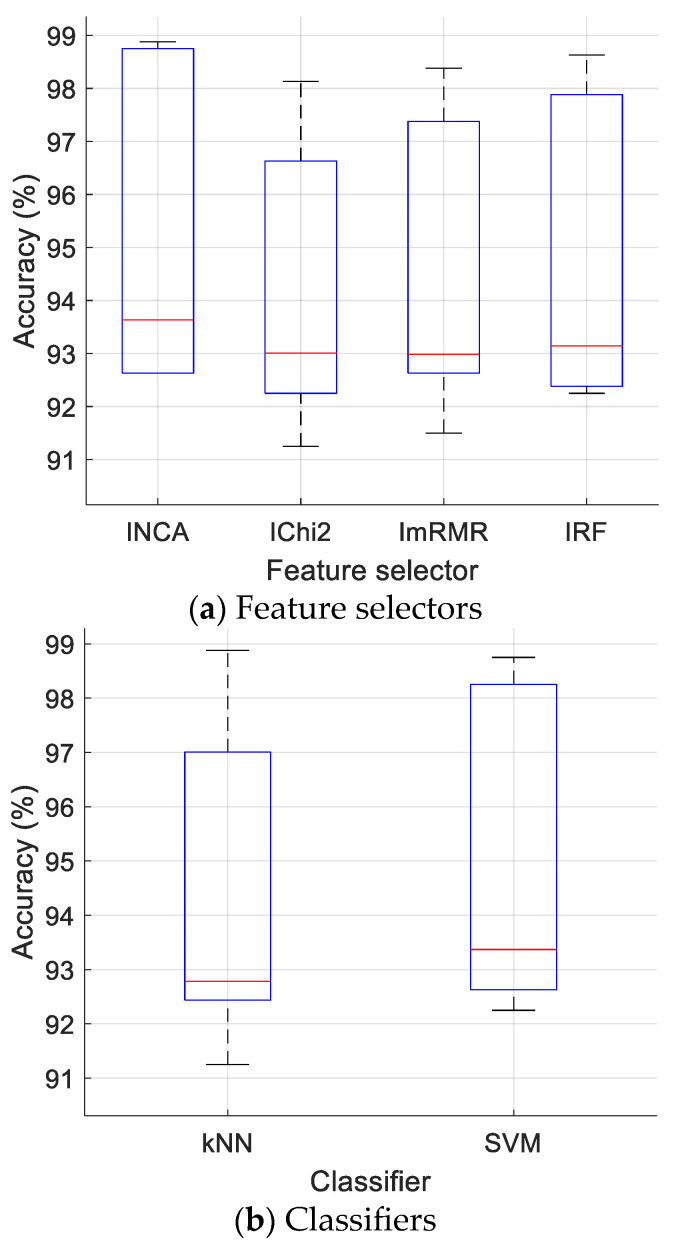
Classification accuracies (%) obtained using various feature selectors and classifiers. The given red lines represent the median of the accuracies.

We evaluated the performance using various feature selectors and classifiers according to Table 4, and the accuracies (%) obtained are shown in Figure 9.

It may be noted from Figure 9 that the best feature selector is INCA, and the best classifier is SVM. Moreover, the voted results are the best results using the various datasets employed. To create the voted results, classifier-based outcomes were used, and the generation methods of these outcomes have been tabulated in Table 5.

**Figure 10:**
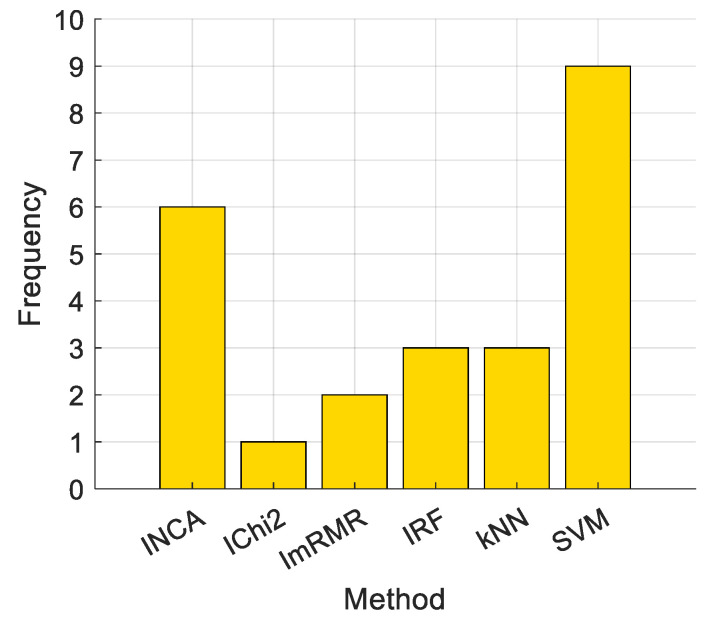
Number of times classifiers and feature selectors were used to obtain the best outcomes.

The results in Figure 9 and Figure 10 pertain to the presented DFE model based on ResNet50. These figures comparatively demonstrate the effectiveness of the feature selectors and classifiers used in the proposed DFE model, providing insights into the robustness and reliability of the results derived from the iterative and voting mechanisms. Figure 8 presents the validation accuracies of the proposed ResNet50* and ResNet50. In this context, the introduced DFE model was implemented using the proposed ResNet50* instead of ResNet50.

Table 5 tabulates a summary of the best-performing methods for various datasets used. The number of times feature selectors and classifiers were used to obtain the best performance by various datasets is depicted in Figure 10.

Figure 10 illustrates that the best-performing feature selector and classifier are NCA and SVM, respectively.

Table 6 compares our developed ResNet50* model with cutting-edge technologies.

Table 6 indicates that our proposed model achieved comparable classification performance of over 92% test classification accuracy for all three image datasets. Only Sharma et al. [52] attained 99.84% classification accuracy by using the ResNet50, but they used five classes and did not select the classes and images from the Kvasir dataset.

The findings, advantages, limitations, and future directions are outlined below.

Findings:

- Developed a ResNet50*-based explainable DFE model that achieved a test performance of more than 92% for all three datasets.
- Employed an IMV technique in the information fusion phase, which significantly enhanced classification outcomes in the form of voted results. This iterative approach, combined with model self-organization, improved decision making.
- Used INCA and SVM as the optimal feature selector and best performing classifier, respectively.
- Generated an attention-based CNN (ResNet50*) model with Grad-CAM, ensuring explainable outcomes with the highest classification performance.
- Presented a DFE model that exhibited a self-organizing characteristic, automatically selecting the best performance outcome.
- In this work, we used four feature selectors to iteratively select features. (i) The INCA is a distance-based feature selector that helps to reduce dimensionality by selecting features based on distance criteria [37]. (ii) The IChi2 selector uses a chi-squared statistical test to measure the independence between features and class labels [53]. (iii) The ImRMR selector aims to minimize redundancy between features while selecting features highly relevant to the target class. This balance is important when many features provide similar information, which may add noise and reduce the interpretability of the model [39]. (iv) IRF is a feature selector that emphasizes distinguishing examples close to the decision boundary, effectively increasing the model’s sensitivity to subtle differences between classes [40].
- kNN is a simple, effective, and widely used distance-based classifier that classifies an example based on the majority label among its k nearest neighbors [54]. SVM is a powerful classifier that works well with high-dimensional spaces and is widely used in image analysis. The combination of kNN and SVM was chosen to balance simplicity and performance.
- The IMV mechanism is a final-stage technique that combines the results obtained from eight classifier-based outcomes (four from kNN and four from SVM) into six aggregated or “voted” outcomes. The aim of IMV is to increase the reliability of the generated outcome by focusing on the consensus between classifiers.
- IMV iterates through the classification results by applying a majority voting scheme to generate more stable, voted outcomes. Using IMV reduces the risk of misclassification due to the weaknesses of any single classifier and produces more generalized results.
- In the final stage, after both classifier-specific and voted outcomes are generated, a greedy algorithm selects the final outcome with the highest classification accuracy. Through this process, the proposed DFE model is transformed into a self-organized DFE model.
- Obtained the best results from the voted outcomes, suggesting the combination of classifiers and feature selectors in a voting scheme.
- Used an attention mechanism in the ResNet50* model that improved the classification performance by focusing on relevant areas within images.

Advantages:

- The proposed ResNet50* combines convolutional residual blocks with pooling-based attention mechanisms. This helps it to focus on important areas in the image and yield highest classification performance.
- High classification accuracy was obtained for all three datasets, demonstrating the generalization of the model.
- The attention-based ResNet50* integrated with Grad-CAM extracts more meaningful features, as it focuses on feature ROI regions.
- The reliability of the proposed DFE model is enhanced by employing IMV, which provides consistent results by reducing the probability of misclassification due to weaknesses in single classifiers. In clinical scenarios, this level of interpretability and reliability is highly desirable and differentiates the model from benchmarks based solely on raw classification outputs. Applying the greedy algorithm with the proposed DFE model transformed it into a self-organized DFE model. Hence, the proposed method is the self-organized DFE model.

Potential clinical applications:

- A new generation explainable ResNet50*-based model is presented as an adjunct tool for radiologists to confirm their findings with high accuracy.
- The heatmaps obtained using Grad-CAM visually explain certain regions that are marked in endoscopy images by the model, enhancing the accuracy and reducing human error.
- The feedback collected on the ROI delineation from clinicians can be used to generate an improved version of the CNN model.
- The presented ResNet50* automatically delineates the visual annotations in reports. Hence, a quick patient record can be obtained.
- Explainable visual outputs help to boost the confidence of clinicians.

Limitations:

- The performance of the model depends on the datasets used, and hence, additional fine-tuning is required while using when using new datasets. Also, if the image quality changes and a new abnormal class is fed as the test data, performance may be affected.
- The ResNet50*-based model contains many parameters due to the convolutional residual blocks and pooling-based attention mechanism (similar to ResNet50), increasing the complexity of the model while also increasing the classification accuracy.

Future directions:

- The proposed ResNet50* can be used for other computer vision applications.
- Integrating ResNet50* with wireless capsule sensors can yield new-generation intelligence sensors.
- The classification capabilities can be further increased by extending ResNet50* to develop other state-of-the-art algorithms.
- Novel adaptive learning mechanisms and optimized models can be proposed to improve decision-making processes.
- The presented model can be employed for other disease diagnoses using various medical images.

## 6. Conclusions

This study introduced a novel ResNet50*-based model designed to enhance the classification capabilities of endoscopy images. We have shown that the classification accuracies were consistently above 92% for various datasets, such as Kvasir, Kvasir v2, and wireless capsule endoscopy (WCE).

The main contribution of this work is the proposed ResNet50* model, developed using convolution-based residual blocks and a pooling-based attention mechanism. Hence, the developed system is able to extract the subtle details from the localized region and obtain the highest classification performance, more than 92% accuracy on all three datasets and 99.13% accuracy using the WCE dataset. The self-organizing DFE approach of our model further enhanced its adaptability and classification accuracy.

We plan to extend this work for the detection of breast cancer, kidney stones, brain tumors, and celiac disease.

## Figures and Tables

**Table 1:** Details of the Kvasir dataset.

No.	Class	Train	Test	Total
1	Dyed lifted polyps	3600	100	3700
2	Dyed resection margins	3600	100	3700
3	Esophagitis	3600	100	3700
4	Normal cecum	3600	100	3700
5	Normal pylorus	3600	100	3700
6	Normal z-line	3600	100	3700
7	Polyps	3600	100	3700
8	Ulcerative colitis	3600	100	3700
Total		28,800	800	29,600

**Table 2:** Summary of classification results (%) obtained using our proposed DFE.

Outcome	Dataset								
	Kvasir			Kvasir v2			WCE		
	Acc	F1	GM	Acc	F1	GM	Acc	F1	GM
C1	92.63	92.60	98.88	98.87	98.87	93.56	98.88	98.87	98.87
C2	91.25	91.24	96.63	96.62	96.59	92.51	96.63	96.62	96.59
C3	91.50	91.42	97.38	97.36	97.34	92.49	97.38	97.36	97.34
C4	92.25	92.23	97.88	97.87	97.85	92.68	97.88	97.87	97.85
C5	92.63	92.62	98.75	98.75	98.74	93.36	98.75	98.75	98.74
C6	92.25	92.26	98.13	98.12	98.11	93.14	98.13	98.12	98.11
C7	92.63	92.62	98.38	98.37	98.37	93.12	98.38	98.37	98.37
C8	92.38	92.38	98.63	98.62	98.61	93.25	98.63	98.62	98.61
V1	92.75	92.74	**99.12**	**99.12**	**99.12**	93.78	**99.12**	**99.12**	**99.12**
V2	**92.87**	**92.87**	98.75	98.75	98.74	93.73	98.75	98.75	98.74
V3	92.50	92.49	**99.13**	**99.12**	**99.12**	**93.92**	**99.13**	**99.12**	**99.12**
V4	92.88	92.86	99	99	98.99	93.69	99	99	98.99
V5	92.88	92.86	98.88	98.87	98.87	93.67	98.88	98.87	98.87
V6	92.75	92.74	98.88	98.87	98.87	93.43	98.88	98.87	98.87

C: Classifier-based outcome, V: Voted outcome, Acc: Classification accuracy, F1: F1-score, GM: Geometric mean. Bold font face highlights the best results.

**Table 3:** Final classification results (%) obtained using our proposed method for different datasets.

Dataset	Accuracy	F1-Score	Geometric Mean
Kvasir	92.87	92.87	92.71
Kvasir v2	94.08	94.08	93.92
WCE	99.12	99.12	99.12

**Table 4:** Feature selector and classifier combination for the proposed DFE.

No.	Feature Selector	Classifier	No.	Feature Selector	Classifier
1	INCA	kNN	5	INCA	SVM
2	IChi2	kNN	6	IChi2	SVM
3	ImRMR	kNN	7	ImRMR	SVM
4	IRF	kNN	8	IRF	SVM

**Table 5:** Summary of best combinations of methods employed for various datasets to yield optimum performance.

Dataset	Generation Method
Kvasir	(INCA and kNN); (INCA and SVM); (ImRMR and SVM); (IRF and SVM)
Kvasir v2	(INCA and kNN); (INCA and SVM); (IRF and SVM); (IChi2 and SVM); (ImRMR and SVM)
WEC	(INCA and kNN); (INCA and SVM); (IRF and SVM)

**Table 6:** Comparison of our work with the state-of-the-art techniques developed for gastrointestinal image classification.

Study	Method	Dataset	Classification Strategies	Results (%)			
				Acc	F1	Pre	Rec
Caroppo et al. [19]	Minimum Redundancy Maximum Relevance, Principal Component Analysis	1. KID2 2. MICCAI 2017, with two classes	10-fold CV	1. 98.20 2. 95.70	1. 98.61 2. 96.43	1. 98.53 2. 97.04	-
Ghosh and Chakareski [20]	AlexNet	1. Capsule endoscopy.org, with two classes 2. KID datasets, with two classes	60:40	1. 94.42 2. 90.69	1. 98.49 2. 88.39	-	-
Zang et al. [21]	Dynamic adversarial adaption network	Collected data, with two classes	4-fold CV	84.02	-	-	-
Lonseko et al. [22]	Attention-Guided CNN	Mixed data (Kvasir, EAD2019, EEC), with ten classes	5-fold CV	93.19	92.80	92.80	92.70
He et al. [23]	DenseNet-121	Collected data, with twelve classes	5-fold CV	91.11	-	-	-
Subedi et al. [24]	DenseNet201	1. GastroVision, with twenty-two classes 2. Kvasir-Capsule, with thirteen classes	5-fold CV	1. 83.86 2. 72.39	1. 83.24 2. 69.00	1. 83.20 2. 70.07	1. 83.86 2. 72.39
Patel et al. [25]	EfficientNet B5	Kvasir, with eight classes	80:20	92.58	93.00	93.00	93.00
Huo et al. [26]	Hierarchical multi-scale feature fusion network	Kvasir, with eight classes	2-fold CV	86.12	86.13	86.25	86.13
Montalbo [46]	Multi-Fused Residual CNN	WCE, with four classes	Hold-out CV 53.3:33.3:13.3	97.75	97.75	-	-
Mohapatra et al. [47]	Empirical wavelet transform + customized CNN	Kvasir, with five classes	Hold-out CV 80:20	88.53	78.87	-	-
Yogapriya et al. [48]	VGG16	Kvasir v2, with seven classes	Hold-out CV 80:20	96.33	96.50	96.50	96.37
Wang et al. [49]	CNN	Kvasir v2, with six classes	Hold-out CV 60:20:20	94.83	94.82	-	-
Noor et al. [50]	CNN	Kvasir v2, with five classes	10-fold CV	96.40	95.24	97.57	93.02
Ramzan et al. [7]	CNN	Kvasir, with eight classes	5-fold CV	95.02	-	94.75	-
Escobar et al. [51]	CNN	Kvasir v2, with five classes	Hold-out CV 80:15:5	98.20	92.76	92.86	92.75
Sharma et al. [52]	ResNet50	Kvasir, with four classes	10-fold CV	99.84	-	-	-
Our model, ResNet50*	DFE based on ResNet50*	Kvasir, with eight classes	10-fold CV	92.87	92.87	-	-
		Kvasir v2, with eight classes	10-fold CV	94.08	94.08	-	-
		WCE, with four classes	10-fold CV	99.12	99.12	-	-

## Data Availability

The authors do not have permission to share data.

## References

[1] Welcome M.O. (2018). Structural and functional organization of the gastrointestinal tract. Gastrointestinal Pyhysiology.

[2] Nightingale J.M., Spiller R. (2023). Normal intestinal anatomy and physiology. Intestinal Failure.

[3] Sandwell M. (2005). The gastrointestinal tract. Developmental Anatomy and Physiology of Children. A Practical Approach.

[4] Li C., Yu W., Wu P., Chen X.D. (2020). Current in vitro digestion systems for understanding food digestion in human upper gastrointestinal tract. Trends Food Sci. Technol..

[5] Cheng L.K., O’Grady G., Du P., Egbuji J.U., Windsor J.A., Pullan A.J. (2010). Gastrointestinal system. Wiley Interdiscip. Rev. Syst. Biol. Med..

[6] Gamage C., Wijesinghe I., Chitraranjan C., Perera I. GI-Net: Anomalies classification in gastrointestinal tract through endoscopic imagery with deep learning. Proceedings of the 2019 Moratuwa Engineering Research Conference (MERCon).

[7] Ramzan M., Raza M., Sharif M., Khan M.A., Nam Y. (2021). Gastrointestinal tract infections classification using deep learning. Comput. Mater. Contin..

[8] Ahmed A. (2022). Classification of gastrointestinal images based on transfer learning and denoising convolutional neural networks. Proceedings of the International Conference on Data Science and Applications: ICDSA 2021.

[9] Jahmunah V., Koh J.E.W., Sudarshan V.K., Raghavendra U., Gudigar A., Oh S.L., Loh H.W., Faust O., Barua P.D., Ciaccio E.J. (2023). Endoscopy, video capsule endoscopy, and biopsy for automated celiac disease detection: A review. Biocybern. Biomed. Eng..

[10] Amina B., Nadjia B., Azeddine B. Gastrointestinal image classification based on VGG16 and transfer learning. Proceedings of the 2021 International Conference on Information Systems and Advanced Technologies (ICISAT).

[11] Su Q., Wang F., Chen D., Chen G., Li C., Wei L. (2022). Deep convolutional neural networks with ensemble learning and transfer learning for automated detection of gastrointestinal diseases. Comput. Biol. Med..

[12] Sharma N., Gupta S., Koundal D., Alyami S., Alshahrani H., Asiri Y., Shaikh A. (2023). U-Net model with transfer learning model as a backbone for segmentation of gastrointestinal tract. Bioengineering.

[13] Kim Y.J., Bae J.P., Chung J.-W., Park D.K., Kim K.G., Kim Y.J. (2021). New polyp image classification technique using transfer learning of network-in-network structure in endoscopic images. Sci. Rep..

[14] Zeng F., Li X., Deng X., Yao L., Lian G. (2021). An image classification model based on transfer learning for ulcerative proctitis. Multimed. Syst..

[15] Rao G.E., Rajitha B., Srinivasu P.N., Ijaz M.F., Woźniak M. (2024). Hybrid framework for respiratory lung diseases detection based on classical CNN and quantum classifiers from chest X-rays. Biomed. Signal Process. Control.

[16] Amer N.S., Belhaouari S.B. (2024). Exploring new horizons in neuroscience disease detection through innovative visual signal analysis. Sci. Rep..

[17] Abad M., Casas-Roma J., Prados F. (2024). Generalizable disease detection using model ensemble on chest X-ray images. Sci. Rep..

[18] Reis H.C., Turk V. (2024). Fusion of transformer attention and CNN features for skin cancer detection. Appl. Soft Comput..

[19] Caroppo A., Leone A., Siciliano P. (2021). Deep transfer learning approaches for bleeding detection in endoscopy images. Comput. Med. Imaging Graph..

[20] Ghosh T., Chakareski J. (2021). Deep transfer learning for automated intestinal bleeding detection in capsule endoscopy imaging. J. Digit. Imaging.

[21] Zhang Y., Zhang K., Ding Y., Liu S., Wang M., Wang X., Qin Z., Zhang X., Ma T., Hu F. (2023). Deep transfer learning from ordinary to capsule esophagogastroduodenoscopy for image quality controlling. Eng. Rep..

[22] Lonseko Z.M., Adjei P.E., Du W., Luo C., Hu D., Zhu L., Gan T., Rao N. (2021). Gastrointestinal disease classification in endoscopic images using attention-guided convolutional neural networks. Appl. Sci..

[23] He Q., Bano S., Ahmad O.F., Yang B., Chen X., Valdastri P., Lovat L.B., Stoyanov D., Zuo S. (2020). Deep learning-based anatomical site classification for upper gastrointestinal endoscopy. Int. J. Comput. Assist. Radiol. Surg..

[24] Subedi A., Regmi S., Regmi N., Bhusal B., Bagci U., Jha D. Classification of Endoscopy and Video Capsule Images Using CNN-Transformer Model. Proceedings of the MICCAI Workshop on Cancer Prevention Through Early Detection.

[25] Patel V., Patel K., Goel P., Shah M. Classification of Gastrointestinal Diseases from Endoscopic Images Using Convolutional Neural Network with Transfer Learning. Proceedings of the 2024 5th International Conference on Intelligent Communication Technologies and Virtual Mobile Networks (ICICV).

[26] Huo X., Sun G., Tian S., Wang Y., Yu L., Long J., Zhang W., Li A. (2024). HiFuse: Hierarchical multi-scale feature fusion network for medical image classification. Biomed. Signal Process. Control.

[27] Islam S., Elmekki H., Elsebai A., Bentahar J., Drawel N., Rjoub G., Pedrycz W. (2023). A comprehensive survey on applications of transformers for deep learning tasks. Expert Syst. Appl..

[28] Yu W., Luo M., Zhou P., Si C., Zhou Y., Wang X., Feng J., Yan S. Metaformer is actually what you need for vision. Proceedings of the IEEE/CVF Conference on Computer Vision and Pattern Recognition.

[29] Selvaraju R.R., Cogswell M., Das A., Vedantam R., Parikh D., Batra D. Grad-cam: Visual explanations from deep networks via gradient-based localization. Proceedings of the IEEE International Conference on Computer Vision.

[30] Nagadia M. Kvasir Dataset. https://www.kaggle.com/datasets/meetnagadia/kvasir-dataset.

[31] Pogorelov K., Randel K.R., Griwodz C., Eskeland S.L., de Lange T., Johansen D., Spampinato C., Dang-Nguyen D.-T., Lux M., Schmidt P.T. Kvasir: A multi-class image dataset for computer aided gastrointestinal disease detection. Proceedings of the 8th ACM on Multimedia Systems Conference.

[32] Montalbo F.J. WCE Curated Colon Disease Dataset Deep Learning. https://www.kaggle.com/datasets/francismon/curated-colon-dataset-for-deep-learning.

[33] Jha D., Kelkalot, Hicks S. VT. Kvasir v2. https://www.kaggle.com/datasets/plhalvorsen/kvasir-v2-a-gastrointestinal-tract-dataset.

[34] He K., Zhang X., Ren S., Sun J. Deep residual learning for image recognition. Proceedings of the IEEE Conference on Computer Vision and Pattern Recognition.

[35] Maillo J., Ramírez S., Triguero I., Herrera F. (2017). kNN-IS: An Iterative Spark-based design of the k-Nearest Neighbors classifier for big data. Knowl. Based Syst..

[36] Vapnik V. (1998). The support vector method of function estimation. Nonlinear Modeling.

[37] Tuncer T., Dogan S., Özyurt F., Belhaouari S.B., Bensmail H. (2020). Novel Multi Center and Threshold Ternary Pattern Based Method for Disease Detection Method Using Voice. IEEE Access.

[38] Tuncer T., Dogan S., Subasi A. (2021). A new fractal pattern feature generation function based emotion recognition method using EEG. Chaos Solitons Fractals.

[39] Tuncer T., Aydemir E., Ozyurt F., Dogan S. (2022). A deep feature warehouse and iterative MRMR based handwritten signature verification method. Multimed. Tools Appl..

[40] Tuncer T., Dogan S., Ozyurt F. (2020). An automated Residual Exemplar Local Binary Pattern and iterative ReliefF based COVID-19 detection method using chest X-ray image. Chemom. Intell. Lab. Syst..

[41] Dogan A., Akay M., Barua P.D., Baygin M., Dogan S., Tuncer T., Dogru A.H., Acharya U.R. (2021). PrimePatNet87: Prime pattern and tunable q-factor wavelet transform techniques for automated accurate EEG emotion recognition. Comput. Biol. Med..

[42] Goldberger J., Hinton G.E., Roweis S., Salakhutdinov R.R. (2004). Neighbourhood components analysis. Adv. Neural Inf. Process. Syst..

[43] Liu H., Setiono R. Chi2: Feature selection and discretization of numeric attributes. Proceedings of the 7th IEEE International Conference on Tools with Artificial Intelligence.

[44] Peng H., Long F., Ding C. (2005). Feature selection based on mutual information criteria of max-dependency, max-relevance, and min-redundancy. IEEE Trans. Pattern Anal. Mach. Intell..

[45] Kononenko I. Estimating attributes: Analysis and extensions of RELIEF. Proceedings of the European Conference on Machine Learning.

[46] Montalbo F.J.P. (2022). Diagnosing gastrointestinal diseases from endoscopy images through a multi-fused CNN with auxiliary layers, alpha dropouts, and a fusion residual block. Biomed. Signal Process. Control.

[47] Mohapatra S., Pati G.K., Mishra M., Swarnkar T. (2023). Gastrointestinal abnormality detection and classification using empirical wavelet transform and deep convolutional neural network from endoscopic images. Ain Shams Eng. J..

[48] Yogapriya J., Chandran V., Sumithra M., Anitha P., Jenopaul P., Suresh Gnana Dhas C. (2021). Gastrointestinal tract disease classification from wireless endoscopy images using pretrained deep learning model. Comput. Math. Methods Med..

[49] Wang W., Yang X., Li X., Tang J. (2022). Convolutional-capsule network for gastrointestinal endoscopy image classification. Int. J. Intell. Syst..

[50] Nouman Noor M., Nazir M., Khan S.A., Song O.-Y., Ashraf I. (2023). Efficient gastrointestinal disease classification using pretrained deep convolutional neural network. Electronics.

[51] Escobar J., Sanchez K., Hinojosa C., Arguello H., Castillo S. Accurate deep learning-based gastrointestinal disease classification via transfer learning strategy. Proceedings of the 2021 XXIII Symposium on Image, Signal Processing and Artificial Vision (STSIVA).

[52] Sharma A., Kumar R., Garg P. (2023). Deep learning-based prediction model for diagnosing gastrointestinal diseases using endoscopy images. Int. J. Med. Inform..

[53] Tasci B., Tasci G., Dogan S., Tuncer T. (2024). A novel ternary pattern-based automatic psychiatric disorders classification using ECG signals. Cogn. Neurodyn..

[54] Zhang S. (2021). Challenges in KNN classification. IEEE Trans. Knowl. Data Eng..

